# Unveiling a groundbreaking alliance: the inaugural collaboration between the Asian Regional Consortium of Headache and *The Journal of Headache and Pain*

**DOI:** 10.1186/s10194-024-01816-0

**Published:** 2024-07-30

**Authors:** Tissa Wijeratne, Surat Tanprawate, Lakshman Singh, Shih-Pin Chen, Paolo Martelletti

**Affiliations:** 1 Western Health, Victoria, VIC Australia; 2 Rajarata University, Mihintale, Sri Lanka; 3 Chiang Mai University, Chiang Mai, Thailand; 4 Victoria University, Victoria, Australia; 5 National Yang Ming University Hospital, Yilan, Taiwan; 6 School of Health, Unitelma Sapienza University, Rome, Italy

The Asia-Oceania region is home to a significant portion of the global population, with diverse demographics across various countries. Recent estimates indicate that the combined population of Asia and Oceania exceeds five billion people, making it the most populous region in the world [1].

Migraine and headache disorders are among the most prevalent neurological disorders worldwide, and their burden in the Asia-Oceania region is substantial [2, 3]. Migraines, tension-type headaches, and other forms of headaches affect over a billion people in this region, impacting their quality of life and productivity [4].

Several factors contribute to the high burden of headaches in Asia and Oceania:

Population Density: Many countries in this region have densely populated urban centers where stress, pollution, and lifestyle factors can contribute to headache disorders.

Socioeconomic Factors: Socioeconomic disparities exist across the region, with some populations lacking access to adequate healthcare resources and facing challenges in effectively managing headache disorders. There are many countries in Asia-Oceania region with no access to headache specialists or a headache training program.

Environmental Factors: Environmental factors, such as air pollution, noise pollution, and climate variations, can trigger or exacerbate headaches in susceptible individuals, particularly in densely populated areas.

Genetic and Ethnic Diversity: The diverse genetic and ethnic makeup of Asian and Oceanian populations may contribute to variations in the prevalence and presentation of headache disorders across different regions and ethnic groups.

Healthcare Infrastructure: Disparities in healthcare infrastructure and access to specialized headache care exist across the region, with some countries facing challenges in providing comprehensive headache management services and even standard migraine therapeutics to their populations.

The Asia-Oceania region faces significant challenges related to the shortage of neurologists and headache specialists as well as the limited availability of headache services and training programs. These challenges contribute to disparities in access to headache care and management, affecting the quality of life of individuals with headache disorders across the region.

Limited Headache Services: Access to specialized headache services, including headache clinics, specialized headache centers, and multidisciplinary treatment programs, is limited in many parts of the region. This lack of dedicated headache services can result in delays in diagnosis, inadequate management of headache disorders, and poor outcomes for individuals with chronic or debilitating headaches.

Inadequate Headache Training Programs: Training programs for healthcare professionals specializing in headache disorders, such as headache medicine fellowships or specialized headache training courses, are not widely available across the Asia-Oceania region. This shortage of training opportunities contributes to a lack of expertise among healthcare providers in effectively diagnosing and managing headache disorders.

Under recognition and Underreporting: Headache disorders are often underrecognized and underreported in many parts of the Asia-Oceania region, leading to a lack of awareness among healthcare professionals, policymakers, and the general public regarding the burden of headache disorders and the need for specialized headache services.

Addressing these challenges requires concerted efforts from healthcare systems, professional organizations, and policymakers to:

Increase the number of neurologists and headache specialists through targeted training programs and incentives to attract healthcare professionals to specialize in headache medicine.

Expand headache services and establish specialized headache centers or clinics to improve access to specialized care for individuals with headache disorders.

Develop and implement headache training programs for healthcare professionals, including neurologists, primary care physicians, nurses, and allied health professionals, to enhance their knowledge and skills in diagnosing and managing headache disorders.

Raise awareness of headache disorders and the importance of specialized headache care among healthcare professionals, policymakers, and the general public to reduce stigma, improve recognition, and promote early intervention for individuals affected by headache disorders.

Collaborative efforts between healthcare providers, professional organizations, patient advocacy groups, and policymakers are essential to address the shortage of neurologists and headache specialists, expand headache services, and improve access to specialized headache care and management programs across the Asia-Oceania region.

Let us reflect on the ninth ARCH meeting, marking a significant milestone with record-breaking attendance from across Asia-Oceania, with over 1450 delegates attending the virtual congress. Despite facing unforeseen challenges and a tight timeline of just four weeks, strong collaboration between Dr. K. Ravishankar and Prof. Tissa Wijeratne transcended borders as they coordinated tirelessly between India and Australia, where their bases were situated. The outcome of this swift coordination was remarkable—a collection of over 50 expertly recorded lectures delivered by leaders in headache medicine. Notably, the distinguished faculty included past, current, and future Presidents of the International Headache Society (IHS) and ARCH, embodying global excellence in the field. While TW oversaw the technical aspects, KR provided invaluable guidance to the organizing team, drawing on his extensive experience and wisdom. Originally expecting a modest attendance of 100–150 experts and trainees, we were astonished to welcome approximately 1450 + delegates—a testament to the event’s resonance within the headache community.

The virtual conference unfolded over two enriching days, featuring comprehensive presentations by esteemed experts, such as Professors David Dodick and Peter Goadsby. KR and TW moderated the sessions and engaged participants in lively discussions, fostering an environment conducive to learning and collaboration. The overwhelming positive feedback we received from delegates underscored the success of the event.

Reflecting on the historic Ninth ARCH2022 Meeting, we recognize the pivotal role of collaboration, cooperation, and communication in its success. By harnessing these synergies, we delivered over 50 high-quality lectures to delegates worldwide, free of charge. Through platforms like www.archhub.org, we disseminated educational materials to 1450 clinicians, advancing headache education and awareness across the region.

Moreover, the active online participation of ARCH members reaffirmed the consortium’s role as a catalyst for training, learning, and discovery in headache medicine. Looking ahead, ARCH is committed to establishing an open-access headache journal, furthering our mission to advance headache care and research in Asia-Oceania.

Asia-Oceania, with its vast population and diverse healthcare landscape, presents unique challenges and opportunities in headache medicine. Since its inception in 2004, ARCH has evolved into a regional powerhouse, uniting nations to promote headache care and awareness. With 19 member countries now under its umbrella, ARCH is poised to expand its reach and impact.

Collaboration remains paramount as we tackle the formidable burden of headache disorders in Asia-Oceania. Through initiatives such as the Asia-HEAD program and virtual series on Inspiring People in Headache Medicine, we aim to inspire the next generation of neurologists and advocate better headache care. The recent WHO-IGAP adoption, the much-awaited WHO IGAP toolkit (to be released in July 2024), underscores the urgent need for continued collaboration and cooperation to improve neurological care and promote brain health [5].

Efforts to address the burden of headaches in the Asia-Oceania region involve raising awareness of headache disorders, improving access to healthcare services, and enhancing research efforts to better understand the underlying causes and effective management strategies. Collaborative initiatives, such as the partnership between the Asian Regional Consortium of Headache and the Journal of Headache and Pain, play a crucial role in advancing knowledge and improving outcomes for individuals affected by headaches in this diverse and populous region.

At this pivotal moment, we are delighted to announce the culmination of a significant partnership between the Asian Regional Consortium of Headaches (ARCH) and the Journal of Headache and Pain. Today marks the commencement of our collaboration, poised to bring forth impactful change in the landscape of headache medicine. As a testament to our joint commitment, we are set to launch our inaugural supplement in conjunction with the 10th ARCH congress. While the Asia-Oceania region has historically lagged behind in the realm of headache medicine, the staggering prevalence of migraines and headache disorders among over a billion individuals in this region demands urgent action. However, amid the shadows cast by these debilitating conditions, a beacon of hope emerges. The pioneering alliance between ARCH and the Journal of Headache and Pain heralds a new dawn, promising a brighter future for those afflicted by migraine and headache disorders in Asia-Oceania.
